# Prediction of potential distributions of *Morina kokonorica* and *Morina chinensis* in China

**DOI:** 10.1002/ece3.11121

**Published:** 2024-03-10

**Authors:** Qing Yuan, Jingjing Zhang, Zhiwen Yao, Quan Zhou, Penghui Liu, Wenhui Liu, Hairui Liu

**Affiliations:** ^1^ College of Eco‐Environmental Engineering Qinghai University Xining China; ^2^ Department of Geological Engineering Qinghai University Xining China; ^3^ State Key Laboratory of Plateau Ecology and Agriculture Qinghai University Xining China

**Keywords:** climate change, MaxEnt, *Morina chinensis*, *Morina kokonorica*, potential suitable habitat

## Abstract

Changes in the habitats of species can provide insights into the impact of climate change on their habitats. Species in the genus *Morina* (Morinoideae) are perennial herbaceous plants that are mainly distributed in the South Asian Mountains and Eastern Mediterranean. In China, there are four species and two varieties of this genus distributed across the Yunnan, Sichuan, Qinghai, and Gansu provinces. This study used the optimal MaxEnt model to simulate past, current, and future potentially suitable habitats of *Morina kokonorica* and *Morina chinensis*. Seventy data of *M. kokonorica* occurrences and 3 of *M. chinensis* were used in the model to predict potentially suitable habitats. The model prediction results indicated that both *M. kokonorica* and *M. chinensis* exhibited trends of northward migration to higher latitudes and westward migration along the Himalayas to higher elevations, suggesting that the northern valleys of Hengduan Mountains and northern and eastern parts of the Himalayas were potential refugia for *M. kokonorica*, and the potential refugia for *M. chinensis* was located in the eastern part of Qinghai‐Tibet Plateau. The results of this niche analysis showed that the two species had higher levels of interspecific competition and that the environmental adaptability of *M. chinensis* was stronger. This research could help further understand the response pattern of *Morina* to environmental change, to understand the adaptability of species to the environment, and promote the protection of species.

## Graphical abstract

We used the optimized Maxent model to simulate the potential habitat areas of *Morina chinensis* and *Morina kokonorica* at different periods, and analyzed the changes in habitat areas, potential refuges, and nichees.

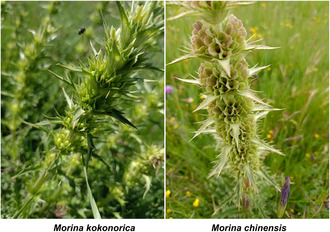

## 1 INTRODUCTION

Global climate change poses marked challenges to human society and ecosystems (Bertrand et al., 2011). According to scientific projections, global warming is expected to persist, resulting in a projected increase of the average surface temperature of the Earth by 0.3–4.5°C by 2100, compared to that during 1986–2005 (Chen, Wang, Chen, & Zhou, 2022; Chen, Wang, Jiang, et al., 2022; Hu et al., 2019; Xiao et al., 2020). The sustainability of global ecosystems is seriously threatened by increasing average temperatures, which have changed the richness of landscapes worldwide (Dawson et al., 2011). Previous studies confirmed that species adapt to warmer environments by relocating to higher elevations or latitudes (Chen et al., 2011; Osland et al., 2021; Paxton et al., 2019; Wiens, 2016). However, the rate of niche change may be slower than that of climate change (Jezkova & Wiens, 2016; Quintero & Wiens, 2013), especially for species restricted to mountaintops or islands where upward migration may be precluded or range shifts may be insufficient to track climatic suitability (Barreto et al., 2021; Wiens, 2016). Hence, low‐dispersal species may face the risk of extinction if future climate change reduces their suitable habitats or if geographic barriers prevent their range expansion (Carlson et al., 2014; Walther et al., 2002). Therefore, assessing potential climatic suitability areas in different periods and population dynamics of species is crucial for biodiversity conservation in future climatic scenarios.

**FIGURE 1:**
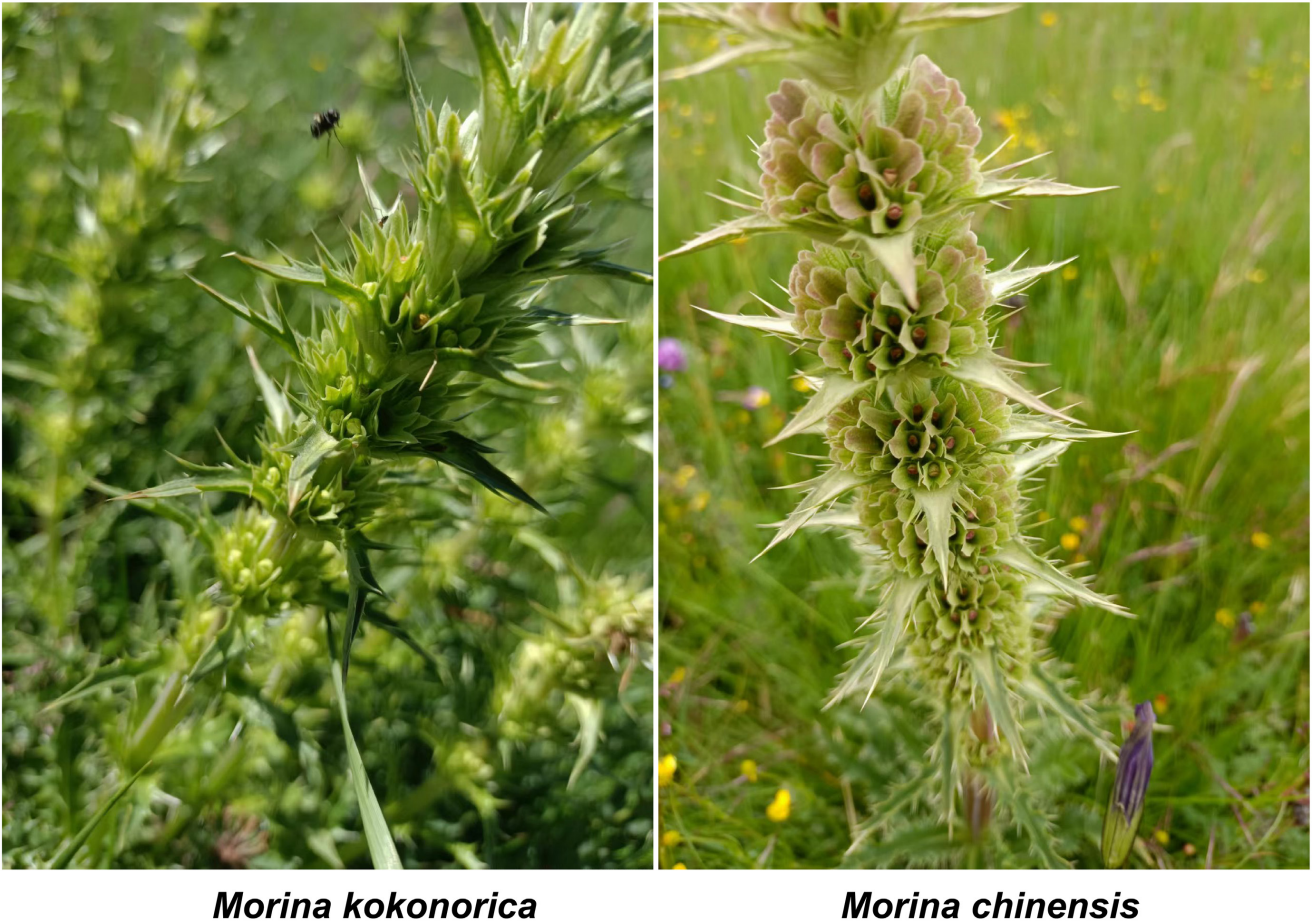
Morphological characteristics of *M. chinensis* and *M. kokonorica*.

The species in the genus *Morina* (Morinoideae) are perennial herbaceous plants that are distributed mainly in the South Asian Mountains, Eastern Mediterranean, and Qinghai–Tibet Plateau. In China, there are four species and two varieties of this genus distributed in the Yunnan, Sichuan, Qinghai, and Gansu provinces. *M. chinensis* has been used as traditional Tibetan medicine to treat joint pain, urinary incontinence, and lower back pain (Su & Takaishi, 1999) and can be found in the center of Gansu, south of Qinghai, west of Sichuan, and west of Inner Mongolia, and it usually grows in the alpine grassy slopes and shrublands at an altitude of about 2800–4000 m. *M. kokonorica* is distributed south of Gansu, Qinghai, northwest of Sichuan, and eastern Tibet and grows on mountain slopes, grasslands, and riverbanks at altitudes of approximately 3000–4500 m. *M. kokonorica* and *M. chinensis* are distributed in the Qinghai‐Tibet Plateau and its surrounding areas, which are closely related to the uplift of the Qinghai‐Tibet Plateau, and the origin and differentiation of the genus *Morina* are significantly associated with the uplift of Himalayan and the retreat of the Tethys Sea according to previous studies (Tang & Li, 1996). *M. kokonorica* and *M. chinensis* both exhibit spiny xeromorphic forms in morphology, which help them adapt to the arid climatic circumstance (Figure 1).

Currently, research on *M. kokonorica* and *M. chinensis* mainly focused on their morphology (Wu et al., 2014) and medicinal value (Teng et al., 2002), but there have been no studies on their distribution or potential refuge. Based on the potential habitat predictions of *M. kokonorica* and *M. chinensis*, we studied the effects of climate change on the habitats of these species and provided a theoretical basis for their rational development and utilization.

Species distribution models have become important tools in ecology and biogeography in recent years (Guisan et al., 2017) and can predict the distribution of species in different periods by combining environmental and species distributions (Elith & Leathwick, 2009; Guo et al., 2017). The MaxEnt model is a shallow machine learning technique (Haneczok & Piskorski, 2020) that has recently gained popularity for ecological predictions. The MaxEnt model is based on the maximum entropy theory and combines data of the occurrences of species as well as bioclimatic and geographic environmental factors to simulate and predict the past, present, and future habitats of species (Phillips et al., 2006). This model has high prediction accuracy, stable prediction results, and good prediction performance even with small datasets of species occurrence, making it the preferred model for the prediction of species habitats (Phillips et al., 2006; Sultana et al., 2020; Wan et al., 2020). The complexity of the MaxEnt model can be managed by setting appropriate regularization multipliers and feature classes (Phillips et al., 2006). Previous studies have shown that the MaxEnt model requires optimization to reduce model overfitting (Morales et al., 2017; Radosavljevic & Anderson, 2014).

In this study, an optimized MaxEnt model was used to simulate the potential species distribution in six periods: the Last Interglacial (LIG), Last Glacial Maximum (LGM), Mid‐Holocene (MH), Current (1970–2000s), and Future (2021–2040, 2041–2060, 2061–2080, 2081–2100). Four shared socioeconomic pathways (SSPs) were included for the Future period. We selected the six most important bioclimatic factors from a total of 19 and three topographic variables (elevation, slope, and aspect) to predict the potential habitats of the two *Morina* species, and explored their potential refugia in the Quaternary glaciation. In addition, the niche breadth, niche overlap, and range overlap of *M. kokonorica* and *M. chinensis* were calculated to explore their interspecific competition.

This study lays the groundwork for future research on the speciation of two *Morina* species and offers valuable insights for their conservation. By elucidating the potential effects of climate change on these species, this study contributes to the development of effective conservation strategies.

## 2 MATERIALS AND METHODS

### 2.1 Species distribution data

**FIGURE 2:**
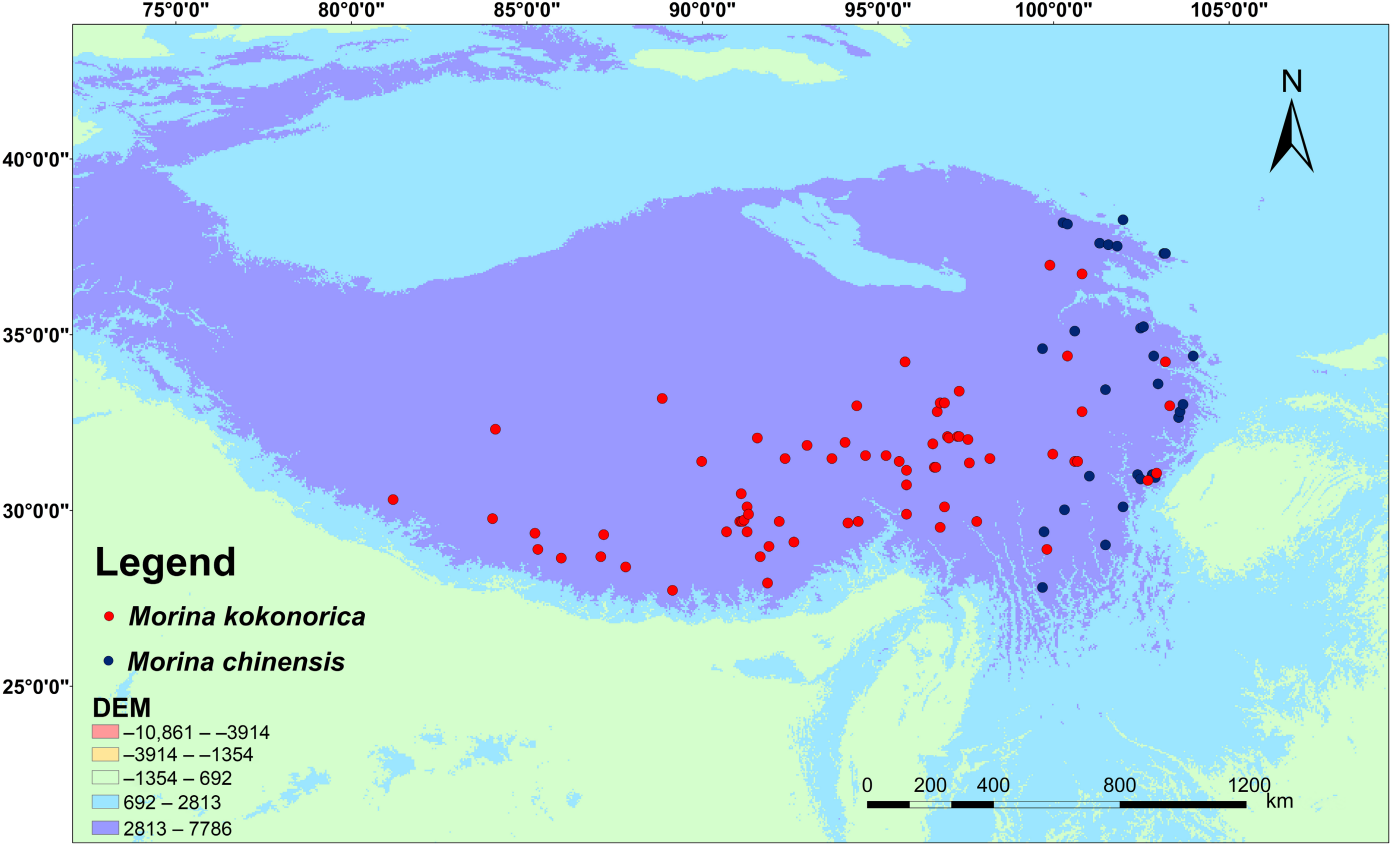
Geographic distribution records of *M. kokonorica* and *M. chinensis*.

The occurrence data for *M. kokonorica* and *M. chinensis* were collected at the Global Biodiversity Information Facility (GBIF; https://www.gbif.org) and the Chinese Virtual Herbarium (CVH; http://www.cvh.ac.cn). In total, data of 116 occurrences of *M. kokonorica* and 36 of *M. chinensis* were obtained after removing duplicate and incorrect loci. To avoid model overfitting caused by data duplication and spatial autocorrelation, ENMTools (Warren et al., 2010) was used to filter the occurrence data using a raster with a spatial resolution of 2.5 arc‐minutes. Finally, data of 70 occurrences of *M. kokonorica* and 30 of *M. chinensis* were retained (Table S1). The final occurrence data are shown in Figure 2.

### 2.2 Environmental data acquisition and screening

Bioclimatic variables were downloaded from the WorldClim v2.1 database (http://www. worldclim.org) (Alemu & Wimberly, 2020; Fick & Hijmans, 2017), and 19 climatic factors were included (Table 1). Future climate data is based on the CMIP6, and the BCC‐CSM2‐MR climate model was selected, which is suitable for the geographical environment of China. This model can well reproduce the global warming trend from 1950 to 2014, as well as the climate variations at different timescales, such as the quasi‐biennial oscillation (QBO) in the equatorial stratosphere, the Madden–Julian Oscillation (MJO), the diurnal cycle of precipitation, the interannual variations of sea surface temperature (SST) in the equatorial Pacific, and the long‐term trend of surface air temperature in the 20th century (Kim et al., 2020). The future data covers four periods (2021–2040, 2041–2060, 2061–2080, and 2081–2100), each with four shared socioeconomic pathways (SSPs).

**TABLE 1:** Description of bioclimatic variables used for MaxEnt model prediction.

Code	Environmental variables	Units
**Bio1**	**Annual mean temperature**	**°C**
Bio2	Mean diurnal range	°C
**Bio3**	Isothermally (BIO2/BIO7) (* 100)	**%**
**Bio4**	Temperature seasonality (standard deviation *100)	**%**
**Bio5**	**Max temperature of warmest month**	**°C**
Bio6	Minimum temperature of coldest month	°C
Bio7	Temperature annual range (Bio5–Bio6)	°C
Bio8	Mean temperature of wettest quarter	°C
Bio9	Mean temperature of driest quarter	°C
**Bio10**	**Mean temperature of warmest quarter**	**°C**
Bio11	Mean temperature of coldest quarter	°C
**Bio12**	**Annual precipitation**	**mm**
Bio13	Precipitation of the wettest month	mm
Bio14	Precipitation of driest month	**mm**
**Bio15**	**Precipitation seasonality (coefficient of variation)**	**°**
Bio16	Precipitation of wettest quarter	mm
Bio17	Precipitation of driest quarter	mm
Bio18	Precipitation of warmest quarter	mm
**Bio19**	**Precipitation of coldest quarter**	**mm**
**Dem**	**Elevation**	**m**
Slope	Slope	**°**
Aspect	Aspect	**°**

*Note*: Bold text indicates the bioclimatic variables used for model construction after screening.

In this study, environmental variable data for the three past periods, the current, and future periods were obtained separately with a spatial resolution of 2.5 arc‐minutes, and the geographic coordinate system was GCS_WGS_1984. The slope and aspect data were extracted using ArcGISv10.4 based on the dem data, which obtained from the Geospatial Data Cloud (https://www.gscloud.cn/home), and the aspect can be divided into four compass directions based on the primary directions: north (315° to 360° and 0° to 45°), east (45° to 135°), south (135° to 225°), and west (225° to 315°) (Liu et al., 2020).

**FIGURE 3:**
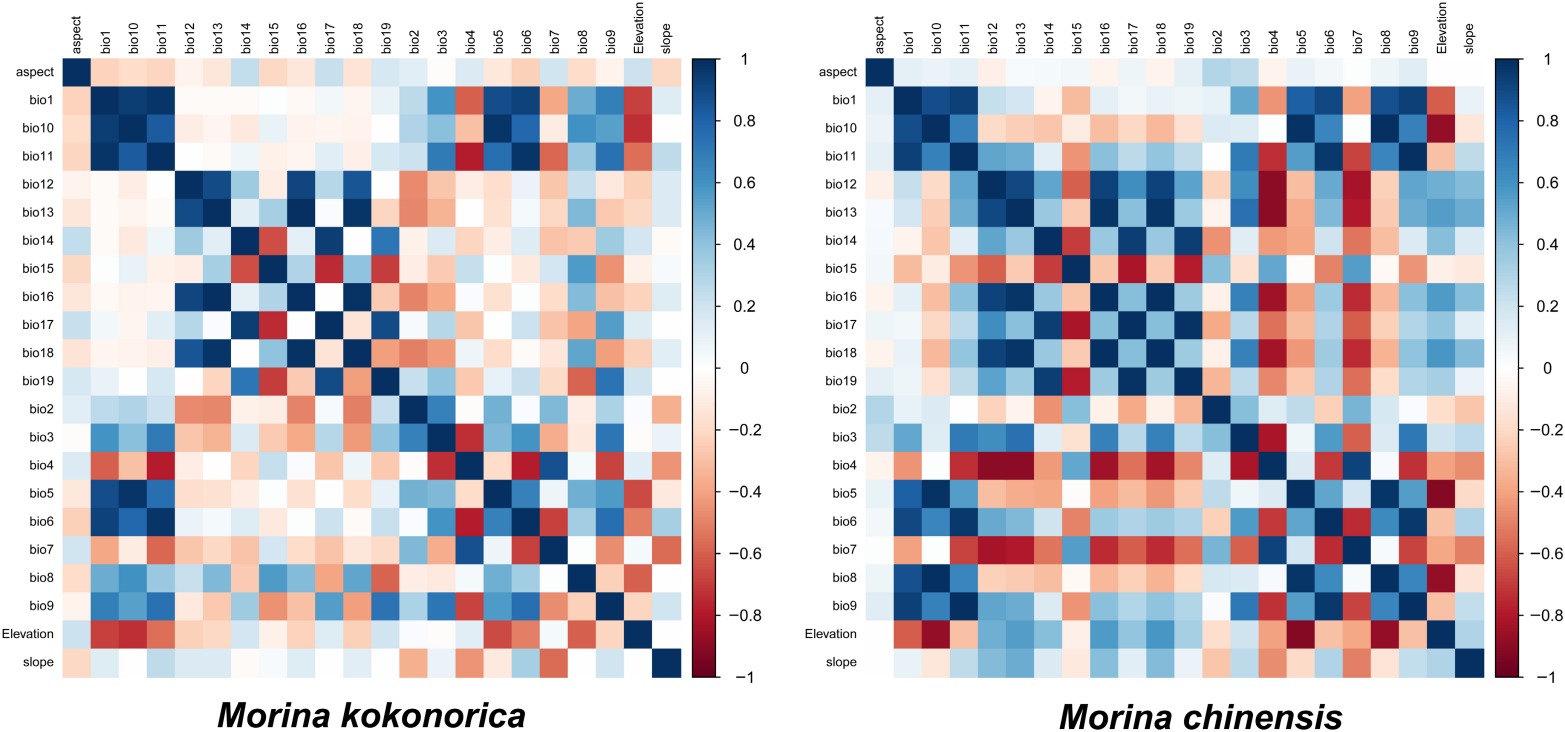
Correlations between 22 environmental variables.

Because a high correlation can easily result in overfitting of the model (Graham, 2003) and reduce the accuracy of the prediction results (Yang et al., 2013), Pearson's correlation coefficients of all bioclimatic variables were evaluated using SPSS v19.0 software. The species occurrence and bioclimatic variables are imported into MaxEnt v3.4.4 software, and obtain the contribution rate and importance of each bioclimatic variable according to the jackknife method. Then, the critical environmental variables were selected by integrating the correlation analysis of all variables and the jackknife estimates of the importance and contribution rate of each variable. For any two highly correlated environmental variables (*r* > .8), we retained the variable that contributed the most to the model. The bioclimatic variables used in this study are listed in Table 1, and the correlations between the bioclimatic variables are shown in Figure 3.

### 2.3 Model construction

**FIGURE 4:**
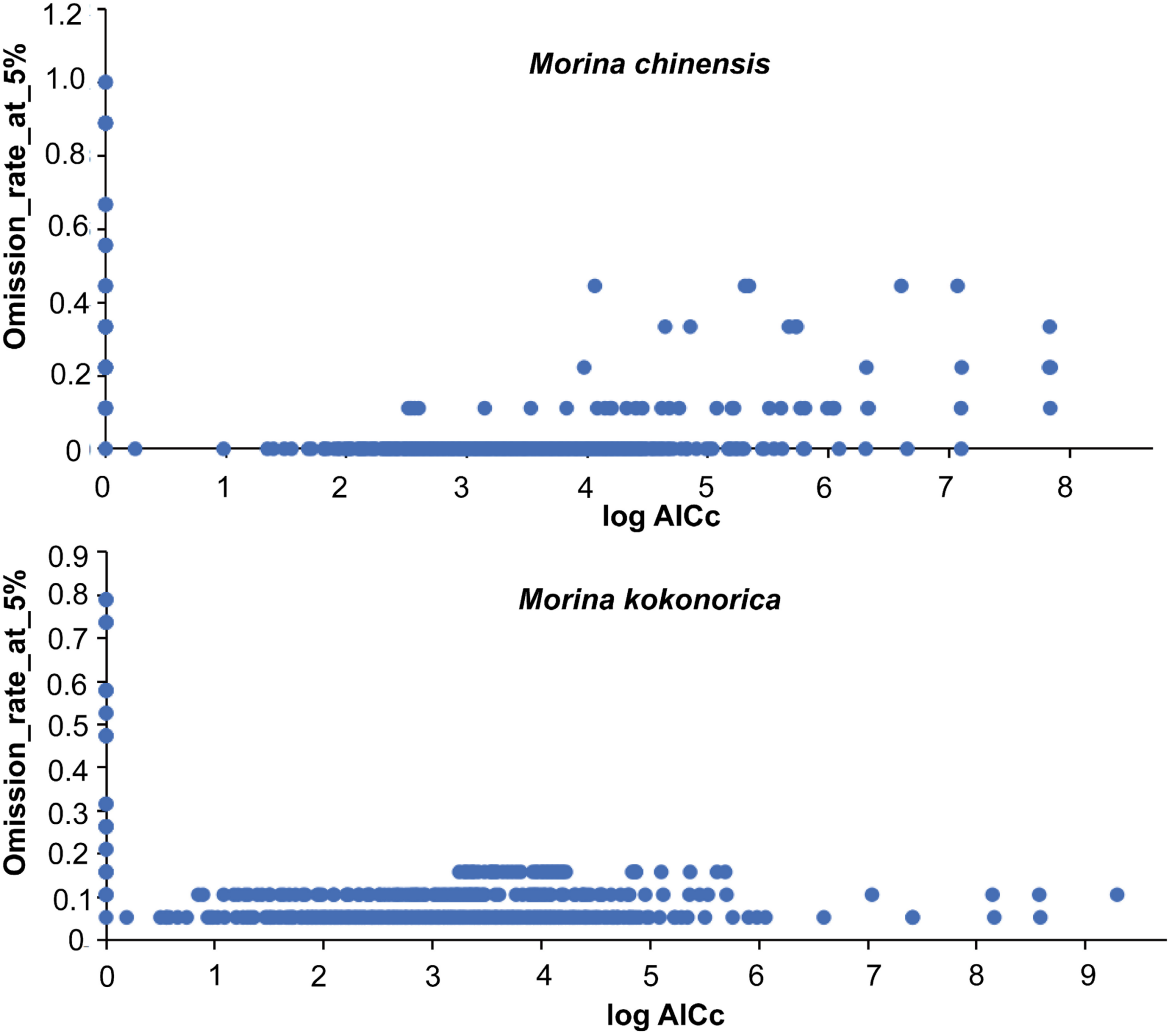
Model optimization results, the results with a data omission rate less than 5% and the minimum delta AICc value were selected.

Previous studies have indicated that the background range of environmental variables used for developing the MaxEnt model can affect the model accuracy and suggested that the model's construction with smaller study areas would result in model overfitting and increase the false‐negative predictions (Amaro et al., 2023). Therefore, this study chose the China range as the study area of environmental variables for model building. The feature classes and regularization multiplier are the most important for the MaxEnt model (Phillips & Dudík, 2008). In this study, the optimal model was selected by evaluating different combinations of feature classes and regularization multipliers. The R script was used to randomly divide the species distribution data into training (75%) and test (25%) sets and the model was built and evaluated (Amiri et al., 2022; Chen, Wang, Chen, & Zhou, 2022; Chen, Wang, Jiang, et al., 2022). An R package “KUENM” was used to optimize the model (Cobos et al., 2019), and we selected the data omission rate of less than 5% and the minimum LogAICc value as the final optimization results (Figure 4).

The final results showed that the optimal selection of feature classes for *M. kokonorica* was linear (L), quadratic (Q), and threshold (T) features, and the regularization multiplier was 1.7. For *M. chinensis*, the optimal selection of feature classes was linear (L) and quadratic (Q) features, and the regularization multiplier was 0.5. In order to reduce the influence of sampling bias on the accuracy of the model, we used 25% of the data as a random test dataset and repeated the model 10 times, and the final simulation result was the mean of 10 repetitions.

The area under the curve (AUC) of the receiver operating characteristic (ROC) curve was calculated to estimate the accuracy of the model (Wiley et al., 2003). It was generally understood that an AUC of less than 0.7 indicated that the low accuracy prediction results of the model could be adopted when the AUC was between 0.7 and 0.9 (Phillips & Dudík, 2008). When the AUC exceeded 0.9, it indicated that the prediction results of this model were highly accurate and could be used for the following analysis (Phillips & Dudík, 2008).

### 2.4 Calculation of niche breadth, niche overlap, and range overlap

Combining the results of the habitat suitability simulation, ENMtools were used to calculate the niche overlap, niche breadth, and range overlap of *M. kokonorica* and *M. chinensis*. For niche overlap, the values of Schoener's D (D) and Hellinger's‐based I (I) (Schoener, 1968; Warren et al., 2008) were calculated, and the range of the values was 0–1, when the value approaches 1, it indicated a higher similarity in the niche. When calculating the range overlap, the threshold was set to 0.2, as areas with a threshold below 0.2 were considered unsuitable habitats.

## 3 RESULTS

### 3.1 Classification of suitable areas

**FIGURE 5:**
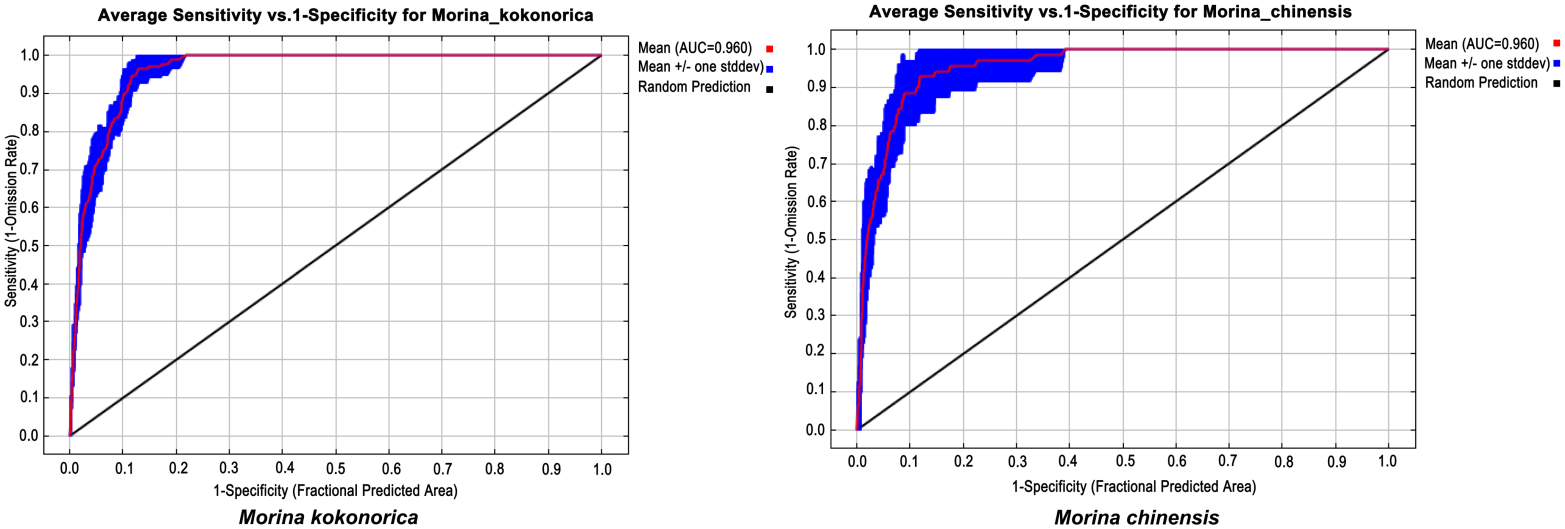
ROC curves of MaxEnt models for *M. kokonorica* and *M. chinensis*.

The AUC values for each period were greater than 0.9 (Figure 5), indicating that the prediction results of this model were highly reliable.

Suitable and unsuitable habitats were reclassified from 0 to 1 using this threshold. The threshold of unsuitable habitats was set at 0.2 based on the findings of the model and the classification standards of prior research; that was, the range of 0.0–0.2 represented an unsuitable area for *M. kokonorica* and *M. chinensis*. Suitable areas were divided into the following grades: lowly suitable areas (0.2–0.4), moderately suitable areas (0.4–0.6), and highly suitable areas (0.6–1.0) (Bao et al., 2022; Ji et al., 2021; Li et al., 2023).

### 3.2 Importance of environmental variables

**FIGURE 6:**
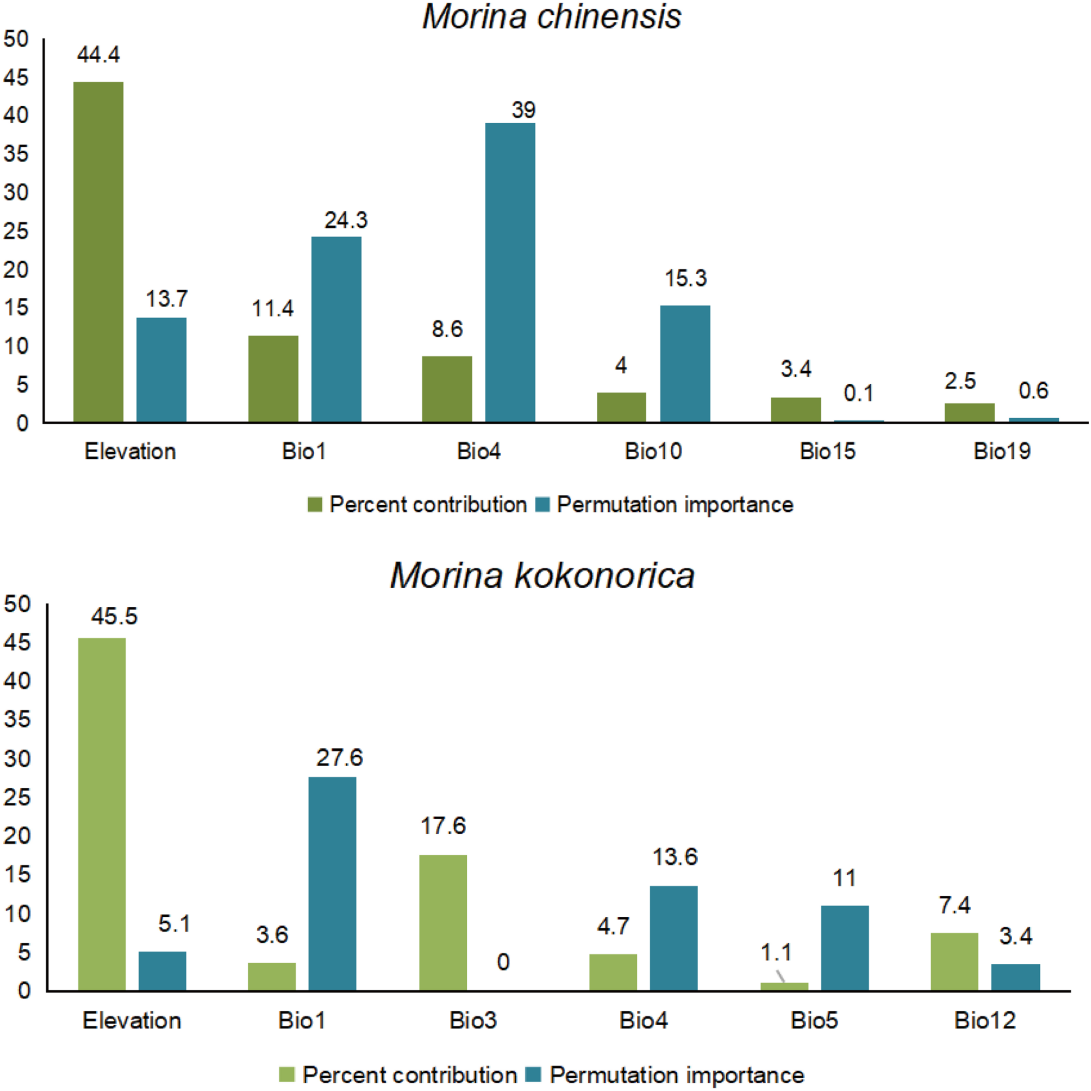
The importance of environmental variables for *M. kokonorica* and *M. chinensis*.

The influential bioclimatic variables used in the MaxEnt model were annual mean temperature (Bio1), isothermally (Bio3), temperature seasonality (standard deviation *100) (Bio4), max temperature of warmest month (Bio5), annual precipitation (Bio12) and elevation for *M. kokonorica*. For *M. chinensis*, the most influential bioclimatic variables were annual mean temperature (Bio1), temperature seasonality (standard deviation *100) (Bio4), mean temperature of warmest quarter (Bio10), precipitation seasonality (coefficient of variation) (Bio15), precipitation of coldest quarter (Bio19) and elevation (Figure 6).

**FIGURE 7:**
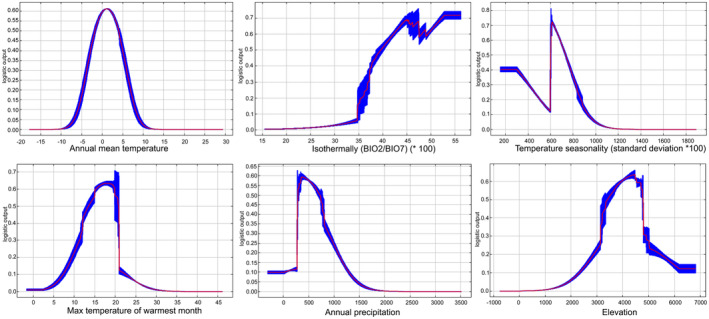
Relationship between the potential distribution probability of *M. kokonorica* and essential environmental factors.

**FIGURE 8:**
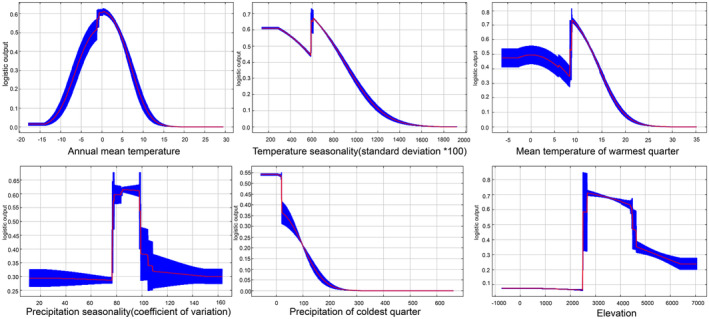
Relationship between the potential distribution probability of *M. chinensis* and essential environmental factors.

To investigate the climatic preferences of *M. kokonorica* and *M. chinensis*, the response curves of the six variables in MaxEnt were analyzed. The results showed that when the value of annual mean temperature was between −3 and 4°C, the isothermal value exceeded 40, the temperature seasonality (standard deviation *100) was between 600 and 750, max temperature of warmest month was between 13 and 20°C, annual precipitation was between 300 and 700 mm, the value of elevation was between 3300 and 4800 m, the probability of the presence of *M. kokonorica* may exceed 50% (Figure 7). For *M. chinensis*, when the value of annual mean temperature was between −3 and 4°C, mean temperature of warmest quarter was between 8 and 13°C, precipitation seasonality (coefficient of variation) was between 68 and 100, precipitation of coldest quarter was lower than 20 mm, the value of elevation was between 2500 and 4500 m, the probability of the presence of *M. chinensis* may exceed 50% (Figure 8).

### 3.3 Species distribution model

#### 3.3.1 Distribution area under current climate

**FIGURE 9:**
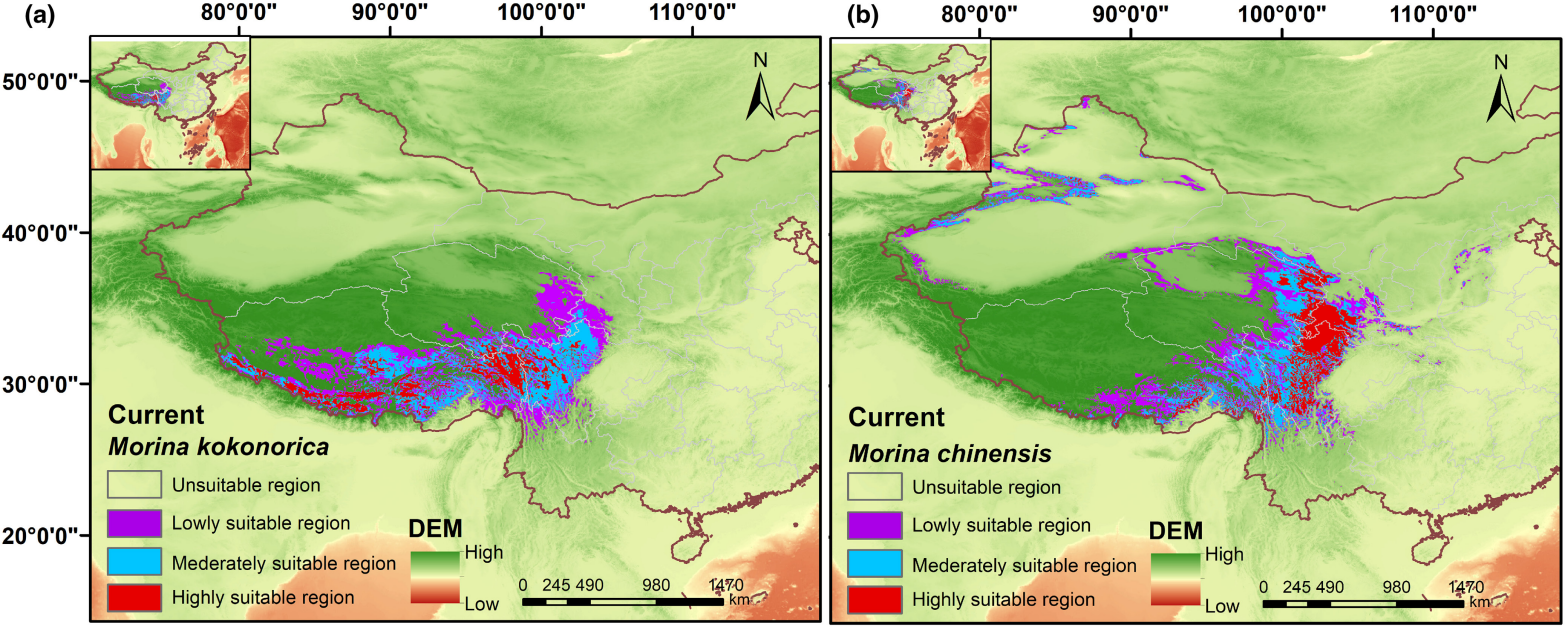
The distribution of the current suitable areas of *M. kokonorica* (a) and *M. chinensis* (b).

Based on the six environmental variables, the current distribution (1970–2000) of *M. kokonorica* and *M. chinensis* was predicted (Figure 9). For *M. kokonorica*, the total area predicted to be suitable was 90.65 × 10^4^ km^2^, the highly suitable area covered 15.50 × 10^4^ km^2^ (17.09%), the moderately suitable area covered 31.17 × 10^4^ km^2^ (34.38%), and the lowly suitable area covered 43.98 × 10^4^ km^2^ (48.51%). For *M. chinensis*, the total area predicted to be suitable was 100.83 × 10^4^ km^2^, the high suitability area covered 21.69 × 10^4^ km^2^ (21.51%), the medium suitability area was 33.13 × 10^4^ km^2^ (32.85%), and the low suitability area was 46.01 × 10^4^ km^2^ (45.63%) (Table 2).

**TABLE 2:** Predicted suitable area in km^2^ for *M. kokonorica* and *M. chinensis*.

Species	Period	Predicted area (×10^4^ km^2^) of the corresponding current area			
		Lowly suitable habitat	Medium suitable habitat	Highly suitable habitat	Total
*Morina kokonorica*	LIG	10.04 (46.13%)	07.79 (35.79%)	03.93 (18.06%)	21.760
	LGM	30.21 (59.80%)	14.12 (27.95%)	06.18 (12.23%)	50.510
	MH	37.24 (61.82%)	16.23 (26.94%)	06.76 (11.22%)	60.230
	Current	43.98 (48.51%)	31.17 (34.38%)	15.50 (17.09%)	90.650
	SSP126 (2021–2040)	44.69 (44.97%)	35.87 (36.10%)	18.80 (18.92%)	99.360
	SSP126 (2041–2060)	42.27 (42.27%)	39.42 (39.42%)	18.31 (18.31%)	100.00
	SSP126 (2061–2080)	43.06 (42.02%)	41.80 (40.79%)	17.61 (17.18%)	102.47
	SSP126 (2081–2100)	44.07 (42.27%)	39.02 (37.42%)	21.16 (20.29%)	104.25
	SSP245 (2021–2040)	42.57 (43.22%)	36.77 (37.33%)	19.14 (19.43%)	98.480
	SSP245 (2041–2060)	44.88 (41.49%)	43.06 (39.80%)	20.23 (18.70%)	108.17
	SSP245 (2061–2080)	46.39 (45.17%)	37.71 (36.72%)	18.59 (18.10%)	102.69
	SSP245 (2081–2100)	46.28 (44.42%)	37.05 (35.56%)	20.85 (20.01%)	104.18
	SSP370 (2021–2040)	42.28 (40.98%)	41.32 (40.05%)	19.57 (18.96%)	103.17
	SSP370 (2041–2060)	45.27 (44.72%)	38.39 (37.93%)	17.55 (17.34%)	101.21
	SSP370 (2061–2080)	43.90 (46.45%)	32.42 (34.30%)	18.19 (19.24%)	94.51
	SSP370 (2081–2100)	48.15 (47.49%)	35.46 (34.98%)	17.76 (17.51%)	101.37
	SSP585 (2021–2040)	46.09 (44.05%)	37.16 (35.51%)	21.37 (20.42%)	104.62
	SSP585 (2041–2060)	51.83 (49.29%)	34.71 (33.00%)	18.61 (17.69%)	105.15
	SSP585 (2061–2080)	48.06 (50.59%)	31.12 (32.76%)	15.81 (16.64%)	94.990
	SS5P85 (2081–2100)	46.40 (48.10%)	32.10 (33.27%)	17.96 (18.61%)	96.460
*Morina chinensis*	LIG	10.19 (52.57%)	06.84 (35.29%)	02.35 (12.12%)	19.380
	LGM	24.74 (46.86%)	24.75 (46.88%)	03.30 (6.25%)	52.790
	MH	36.80 (57.85%)	22.84 (35.90%)	03.97 (6.24%)	63.610
	Current	46.01 (45.63%)	33.13 (32.85%)	21.69 (21.51%)	100.83
	SSP126 (2021–2040)	83.09 (53.26%)	43.30 (27.75%)	29.61 (18.98%)	156.00
	SSP126 (2041–2060)	75.56 (51.37%)	40.85 (27.77%)	30.66 (20.84%)	147.07
	SSP126 (2061–2080)	77.27 (52.35%)	41.59 (28.17%)	28.74 (19.47%)	147.60
	SSP126 (2081–2100)	80.93 (52.95%)	43.04 (28.35%)	27.84 (18.33%)	151.81
	SSP245 (2021–2040)	81.60 (54.98%)	40.67 (27.40%)	26.14 (17.61%)	148.41
	SSP245 (2041–2060)	69.15 (51.02%)	39.30 (28.99%)	27.07 (19.97%)	135.52
	SSP245 (2061–2080)	70.12 (51.69%)	37.74 (27.82%)	27.77 (20.47%)	135.63
	SSP245 (2081–2100)	76.78 (53.80%)	39.42 (27.62%)	26.51 (18.57%)	142.71
	SSP370 (2021–2040)	79.31 (53.69%)	42.52 (28.25%)	28.68 (19.05%)	150.51
	SSP370 (2041–2060)	79.08 (52.97%)	40.88 (27.38%)	29.33 (19.64%)	149.29
	SSP370 (2061–2080)	75.70 (51.93%)	42.56 (29.20%)	27.49 (18.86%)	145.75
	SSP370 (2081–2100)	74.29 (51.23%)	40.18 (27.70%)	30.54 (21.06%)	145.01
	SSP585 (2021–2040)	73.66 (52.49%)	39.67 (28.27%)	26.99 (19.23%)	140.32
	SSP585 (2041–2060)	69.89 (50.49%)	39.99 (28.89%)	28.52 (20.60%)	138.40
	SSP585 (2061–2080)	78.96 (52.60%)	42.15 (28.07%)	29.00 (19.31%)	150.11
	SS5P85 (2081–2100)	77.57 (51.55%)	42.20 (28.04%)	30.68 (20.39%)	150.45
Overlapped areas	Current	42.77			

In this study, *M. kokonorica* was distributed mainly in the southern Qinghai Province, the Himalayan Mountains, and the northern Hengduan Mountains (Figure 9a). Suitable habitats of *M. chinensis* were distributed mainly in eastern Qinghai Province, northwestern Sichuan Province, northern parts of Himalayan Mountains, northern Hengduan Mountains, and southern Qilian Mountains (Figure 9b). The distribution of suitable areas for the two species that were simulated by the MaxEnt model was generally consistent with that obtained from the field survey and the recorded specimens.

#### 3.3.2 Prediction of suitable areas in the historical period

**FIGURE 10:**
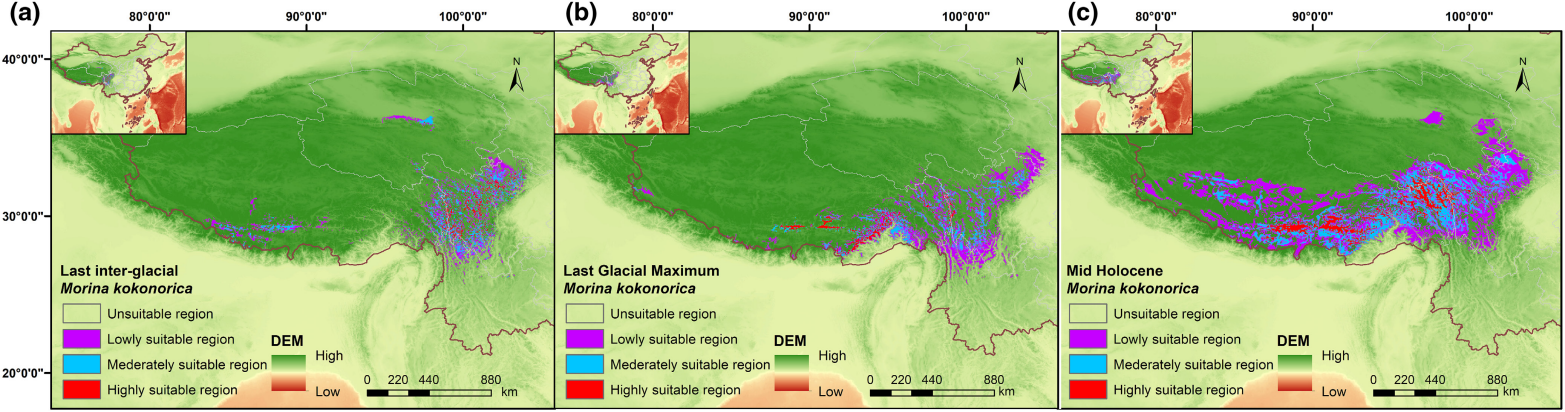
The suitable areas in the past three periods (a, LIG; b, LGM; and c, MH) for *M. kokonorica*.

According to the simulation of the suitable areas for *M. kokonorica* for the past three periods (LIG, LGM, and MH) (Figure 10), the suitable areas were distributed mainly in the Hengduan Mountains, and the highly suitable area covered 3.93 × 10^4^ km^2^ in the last interglacial period (Figure 10a). During the last glacial maximum (Figure 10b), the highly suitable area was 6.18 × 10^4^ km^2^ and was distributed mainly in the Hengduan Mountains. In the MH period (Figure 10c), the highly suitable area had already expanded to 6.76 × 10^4^ km^2^ (Table 2).

**FIGURE 11:**
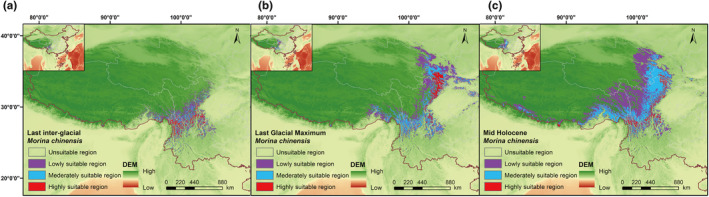
The suitable areas in the past three periods (a, LIG; b, LGM; and c, MH) for *M. chinensis*.

The highly suitable areas for *M. chinensis* were approximately 2.35 × 10^4^ km^2^ in the last interglacial period (Figure 11a), 3.30 × 10^4^ km^2^ in the last glacial maximum period (Figure 11b), and 3.97 × 10^4^ km^2^ in the MH period (Figure 11c; Table 2). Therefore, highly suitable areas for *M. chinensis* were deemed to have gradually expanded (Figure 11). Analysis of the simulation results for suitable habitats from the three past periods revealed a notable expansion of highly suitable areas. Furthermore, these areas exhibited a distinct northward migration trend originating from the Hengduan Mountains.

#### 3.3.3 Future potential distribution

**FIGURE 12:**
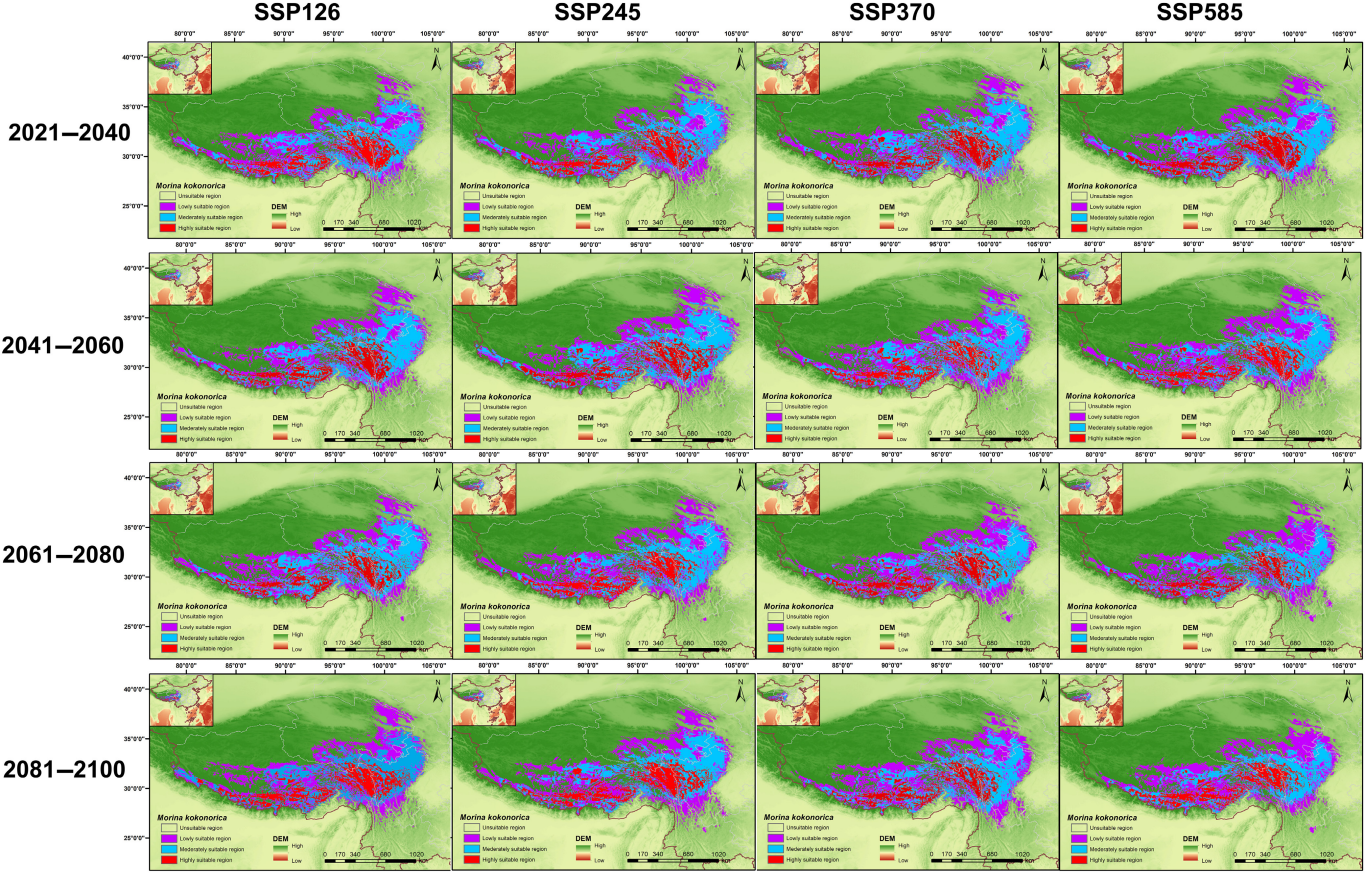
Potential suitable habitat of *M. kokonorica* under future climatic conditions.

**FIGURE 13:**
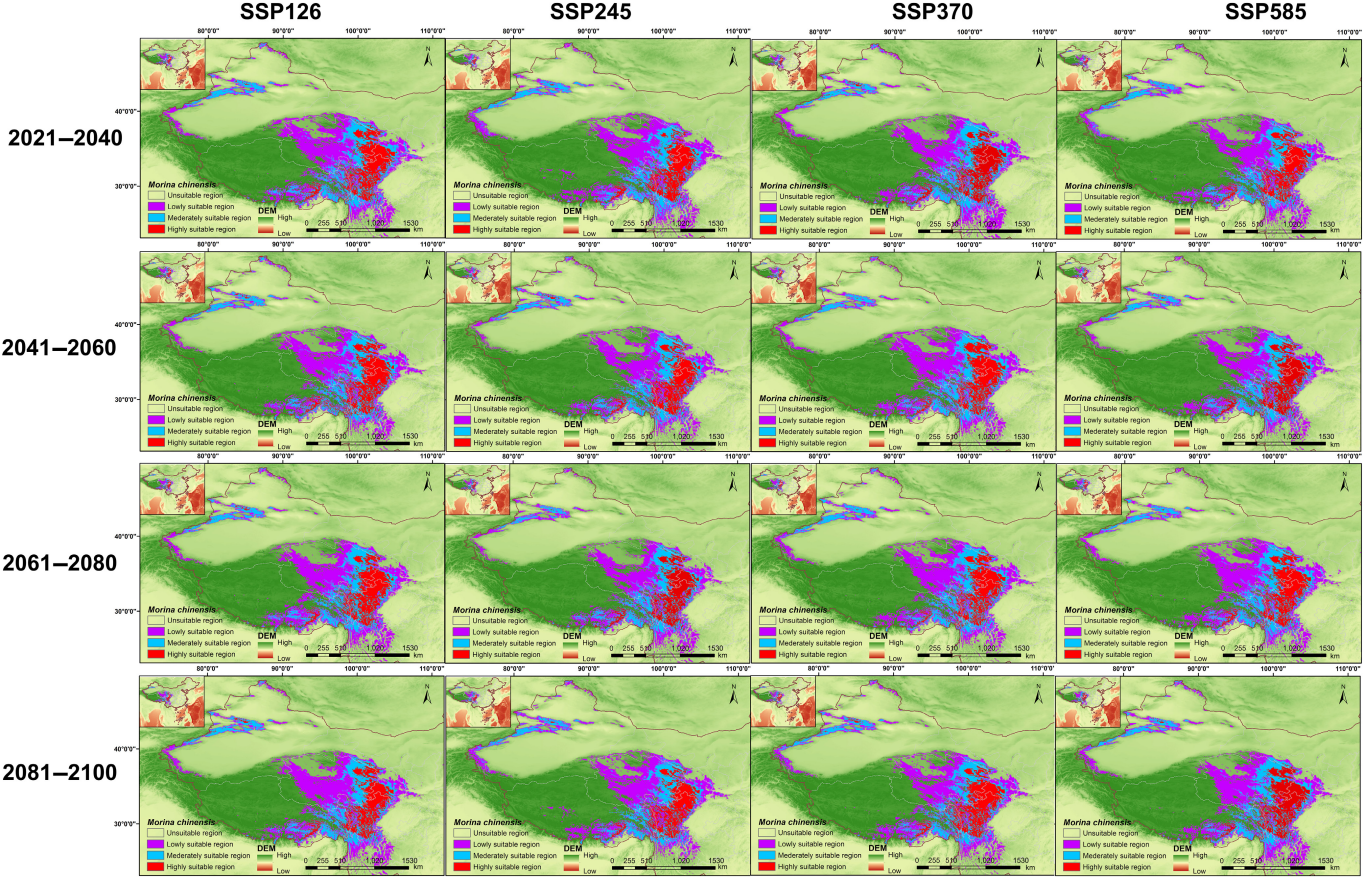
Potential suitable habitat of *M. chinensis* under future climatic conditions.

The potentially suitable habitats of *M. kokonorica* and *M. chinensis* under four shared socioeconomic pathways (SSPs) in four periods (2021–2040, 2041–2060, 2061–2080, and 2081–2100) were visualized using ArcGIS (Figures 12 and 13).

For *M. kokonorica*, the high suitability area was distributed mainly in the Himalayas, southern Qinghai Province, and northern Hengduan Mountains (Figure 12). Under the SSP126 scenario, the total suitable habitat area increased from 99.36 × 10^4^ to 104.25 × 10^4^ km^2^, and the high suitability area increased from 18.80 × 10^4^ km^2^ in the 2021–2040 period to 21.16 × 10^4^ km^2^ in the 2081–2100 period. Under the SSP585 scenario, compared with the 2041–2060 period, the total suitable habitat area decreased by 10.16 × 10^4^ km^2^ in the 2061–2080 period. In the 2021–2040 period, the highest proportion of high suitability area was under the SSP585 scenario, while in the 2040–2060 period, the highest proportion of high suitability area was under the SSP245 scenario (Table 2).

For *M. chinensis*, the high suitability areas were mainly distributed in the Hengduan Mountains, eastern Qinghai Province, and southern Gansu Province (Figure 13). Under the SSP370 scenario, the total suitable habitat area gradually decreased, the percentage of low suitability area also gradually decreased, but the medium suitability area remained stable, and the high suitability area increased in the 2081–2100 period. Under the SSP245 scenario, compared with the 2021–2040 period, the total suitable habitat area decreased by 12.89 × 10^4^ km^2^ in the 2041–2060 period, but the high suitability area increased. Under the SSP585 scenario, the high suitability area and the medium suitability area gradually increased, but the low suitability area fluctuated within a certain range. Compared with the present period, the simulation results for the future periods indicate that the total suitable habitat area of *M. chinensis* was expanded, mainly in its low suitability area (Table 2).

#### 3.3.4 The overlap region of *M. kokonorica* and *M. chinensis* under the current period

**FIGURE 14:**
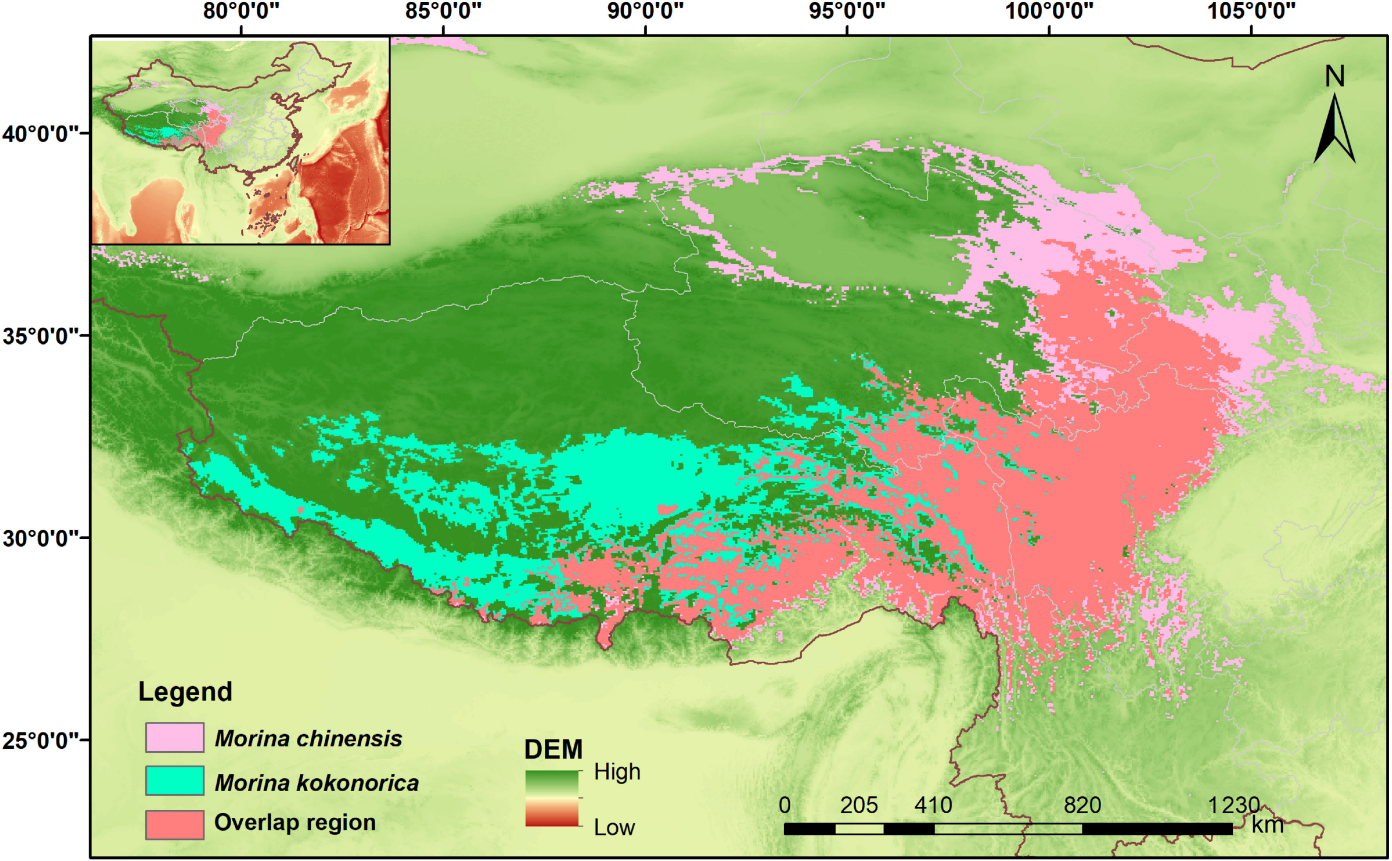
Overlapped suitable habitat of two species of *Morina*.

The overlap of suitable areas for *M. kokonorica* and *M. chinensis* under the current period is shown in Figure 14. The overlapping areas were 42.77 × 10^4^ km^2^ and were distributed mainly in eastern Qinghai Province, northwestern Sichuan Province, northern Hengduan Mountains, and the northern parts of Himalayan Mountains. It is suggested that *M. kokonorica* and *M. chinensis* have a large overlap of suitable habitat areas in the present period, which is consistent with our field survey results that two species were located in the same area. This indicates that the *M. kokonorica* and *M. chinensis* are sympatric in some regions, and there is no strict geographic isolation between the two species.

#### 3.3.5 Niche analysis of *M. kokonorica* and *M. chinensis*

The niche overlap of *M. kokonorica* and *M. chinensis* was high, with a D value of 0.6267 and an I value of 0.87374 (Table 3). The rate of range overlap between the two species was 0.66073, indicating a high degree of habitat overlap. The niche breadth of *M. chinensis* was 0.8873, whereas that of *M. kokonorica* was 0.8755. The niche breadth of *M. chinensis* was higher than that of *M. kokonorica*.

**TABLE 3:** Niche analysis of *M. kokonorica* and *M. chinensis*.

Niche overlap		Range overlap	Niche breadth
D	I		
0.6267	0.87374	0.66073	0.8755/0.8873

## 4 DISCUSSION

*M. kokonorica* and *M. chinensis* were distributed mainly in and around the Qinghai–Tibet Plateau. In this study, the occurrence data of *M. kokonorica* and *M. chinensis* were collected by GBIF and CVH. The suitable habitats of the two species were simulated under historical, current, and future climatic scenarios, and their responses to climate change were deduced. This information was then used to elucidate interspecific competition and migration. Furthermore, this study lays the foundation for ecological restoration and environmental conservation in the Qinghai–Tibet Plateau and its adjacent regions.

### 4.1 Influence factors of simulation accuracy

Despite the widespread use of MaxEnt models, unoptimized models can lead to inaccurate predictions (Kong et al., 2019). Although there are some options for model improvement in the MaxEnt software, there is no universally agreed‐upon method for selecting parameters (Syfert et al., 2013). As earlier research indicates, a significant proportion (87%) of MaxEnt model experiments have utilized data that are subject to sampling bias (Yackulic et al., 2013). This can compromise the predictive ability of the model and result in overfitting, thus limiting the accuracy and reliability of the results (Araújo & Guisan, 2006; Kadmon et al., 2004; Reese et al., 2005).

Moreover, the accuracy of the final result was also influenced by the complexity of the MaxEnt model, which was influenced mainly by three factors: the number of environmental variables modeled, feature classes (feature options in the software), and the regularization multiplier. In this study, the occurrence data were screened using “ENMtools”, and the selection of environmental variables was combined with the importance of the pre‐experimental results through SPSS correlation analysis. The feature classes and regularization multiplier were optimized and selected through the R package “KUENM”. Hence, the prediction results of the model used in this study are highly reliable.

### 4.2 Distribution pattern and potential refugia

The interaction between the climate and plants has been a popular topic in ecology, geography, and meteorology (Dusenge et al., 2019). Climate change has a profound effect on species distribution, and changes in species distribution may reflect climate change. For instance, global warming has brought the distribution of herbaceous plants to higher elevations (Chen et al., 2011; He et al., 2019; Parmesan & Yohe, 2003), but this warming trend may also threaten species that originally lived in high mountainous areas, thereby reducing their distribution (Chen et al., 2011; Quintero & Wiens, 2013). *M. kokonorica* and *M. chinensis* were distributed mainly in high‐altitude areas and may be substantially affected by climate change. Based on the simulation results from the three past periods, it can be observed that both *M. kokonorica* and *M. chinensis* have exhibited trends of not only northward migration to higher latitude but also westward migration along the Himalayas to higher elevations. This observation is consistent with the findings of most studies on suitable habitats (Anand et al., 2021; Yan et al., 2021; Yang et al., 2022). Comparing the simulation results of potentially suitable areas between the current and future periods, there was little change in total suitable areas for *M. kokonorica*. This may be due to the fact that plants require a longer time to adapt to climate change through migration to higher altitudes, while environmental changes occur at a more rapid pace. The total suitable areas of *M. chinensis* would increase a lot in the future, but the areas of its high suitability areas would change a little, so we infer that *M. chinensis* has stronger environmental adaptability and resistance to environmental changes, and niche analysis also shows that *M. chinensis* has stronger environmental adaptability.

Research on potential refuges during the Quaternary Glacial period has marked implications for our understanding of the current plant distribution patterns and future evolution (Dai et al., 2022). The MaxEnt model was used to predict the distribution in different periods and thus infer potential refuges (Chen et al., 2011). In this study, the prediction results indicated that the northern parts of Hengduan Mountains and northern and eastern parts of Himalayas were the potential refugia of the Last Glacial Maximum period for *M*. *kokonorica*, which is consistent with the previous conclusion that the Hengduan Mountains are the typical plant refugia (Ding et al., 2020; Sun et al., 2017), and the potential refugia for *M*. *chinensis* were located in the east of Qinghai‐Tibet Plateau. Based on the refugium of the two species in the Last Glacial Maximum period, the dispersal routes of the two species may partially overlap after the end of the glacial period, which could explain that the two species are sympatric distribution in some areas.

### 4.3 The niche of *M. kokonorica* and *M. chinensis*

Niche overlap is an indicator of ecological similarity between species and is related to interspecific competition. Generally, a high degree of niche overlap implies high similarity in resource utilization between species and more severe competition (Russel et al., 2017; Xiong et al., 2022). In this study, a high degree of niche overlap indicated a high level of interspecific competition between these two species. The niche breadth of *M. chinensis* was higher than that of *M. kokonorica*, indicating that *M. chinensis* has stronger environmental adaptability and greater competitiveness in its sympatric distribution with *M. kokonorica*. Based on the results of the environmental factor selection, it was determined that there are both similarities and differences in the preferences of *M. chinensis* and *M. kokonorica*. Annual mean temperature (Bio1) has a large influence on the potential distribution of both species, but the model simulation results show that the optimal annual mean temperature for *M. kokonorica* is 2°C, while that for *M. chinensis* is around 0°C. Although both species are mainly distributed in the Qinghai‐Tibet Plateau and its surrounding high‐altitude areas, there is a large difference in the altitude of their distribution. *M. kokonorica* tends to grow at altitudes of 3300–4800 m, while *M. chinensis* prefers to grow at 2500–4500 m. In addition, *M. kokonorica* starts to bloom in June, while *M. chinensis* starts to bloom in July. The apex of the calyx for *M. chinensis* is rounded, while the apex of the calyx for *M. kokonorica* is usually spinose. The leaves of *M. chinensis* are shallowly lobed, and the leaves of *M. kokonorica* is cleft almost to midvein. Thus, the spatial–temporal heterogeneity and morphological differentiation between *M. kokonorica* and *M. chinensis* may be the reason why the two species can coexist in some areas as two separate species.

*M. kokonorica* and *M. chinensis* have such a large niche overlap, and the two species are sympatric in some areas, indicating that there is no complete geographic isolation between the two species. Therefore, we infer that two species may be in the process of divergence, and there may be gene flow between them. However, the specific divergence process needs to be verified by subsequent population genetic studies.

### 4.4 Suggestion on wild population protection of *M*. *kokonorica* and *M*. *chinensis*

This study found that under future climate scenarios, except for the expansion of the low suitability area of *M. chinensis*, the potential habitats of two species of *Morina* are with little changes, but it does not mean they would not be under the threat of their survival in the future. The ecosystem of the Qinghai–Tibet Plateau is highly susceptible to human activities (Wei et al., 2022) and this study does not consider the influence of human factors. Therefore, establishing ecological barriers to protect this species in areas with high habitat suitability is crucial. Additionally, the establishment of resource reserves and wild‐plant observation stations can facilitate the conservation of these species and their habitats. By implementing these measures, we could work toward ensuring the long‐term survival and persistence of these species in the face of environmental changes.

## 5 CONCLUSION

In this study, an optimized MaxEnt model was used to simulate the potential species distribution of *M. kokonorica* and *M. chinensis*. The simulation results indicated that *M. kokonorica* and *M. chinensis* have shown a northward migration trend, which is consistent with the responses of the species to climate change in previous studies. The northern valleys of Hengduan Mountain and northern and eastern parts of Himalayas were the refuge for *M. kokonorica*, and the potential refugia for *M*. *chinensis* were located in the east of Qinghai‐Tibet Plateau. The investigation of potential plant refugia during the Quaternary glaciation has key implications for our understanding of current plant distribution patterns and their future evolution. The niche overlap calculation results showed that *M. kokonorica* and *M. chinensis* had high niche overlap, indicating high interspecific competition between the two species. Compared with *M. kokonorica*, the niche breadth of *M. chinensis* was higher, indicating that *M. chinensis* has a higher level of interspecific competition. High niche overlap and range overlap indicate that there is no complete geographic isolation between the two species, and the gene flow may be occurred in it, so this study suggests that these two species may be in the process of divergent evolution. By elucidating the mechanisms of which plants adapt to climatic change, this study provides valuable insights into the potential impacts of future climate change on plant communities and informs the development of effective conservation strategies.

## AUTHOR CONTRIBUTIONS

**Qing Yuan:** Conceptualization (equal); visualization (equal); writing – original draft (equal). **Jingjing Zhang:** Validation (equal). **Zhiwen Yao:** Resources (equal). **Quan Zhou:** Methodology (equal). **Penghui Liu:** Investigation (equal). **Wenhui Liu:** Conceptualization (equal). **Hairui Liu:** Writing – review and editing (equal).

## FUNDING INFORMATION

CAS Light of West China Program (xbzglzb2022043); National Natural Science Foundation of China: Speciation of genus *Triosteum* (Caprifoliaceae) in China (grant number 32260059); Joint Grant from Chinese Academy of Sciences and People's Government of Qinghai Province on Sanjiangyuan National Park (LHZX‐2021‐04).

## Supporting information


Table S1.

## Data Availability

The data supporting the findings of this study were obtained from the WorldClim (https://worldclim.org/), Geospatial Data Cloud (https://www.gscloud.cn/home), Chinese Virtual Herbarium (https://www.cvh.ac.cn/), and GBIF (https://www.gbif.org/) databases. All data sources complied with legal requirements.
