# Supplementary material for: Prediction of potential distributions of Morina kokonorica and Morina chinensis in China
Source: Ecol Evol. 2024 Mar 10;14(3):e11121. doi: 10.1002/ece3.11121 (PMC10925826; doi:10.1002/ece3.11121)
Supplement: Supplementary file 1 — Table S1. [file ECE3-14-e11121-s001.docx]

TABLE S1 The occurrence data for *M. kokonorica* and *M. chinensis*.

| **Species** | **Longitude** | **Latitude** | **Source** |
| --- | --- | --- | --- |
| *Morina chinensis* | 100.2708 | 38.1875 | CVH |
| *Morina chinensis* | 100.3125 | 30.02083 | CVH |
| *Morina chinensis* | 100.3958 | 38.14583 | CVH |
| *Morina chinensis* | 100.6042 | 35.10417 | CVH |
| *Morina chinensis* | 101.0208 | 30.97917 | GBIF |
| *Morina chinensis* | 101.3125 | 37.60417 | GBIF |
| *Morina chinensis* | 101.4792 | 29.02083 | GBIF |
| *Morina chinensis* | 101.4792 | 33.4375 | GBIF |
| *Morina chinensis* | 101.5625 | 37.5625 | CVH |
| *Morina chinensis* | 101.8125 | 37.52083 | GBIF |
| *Morina chinensis* | 101.9792 | 30.10417 | CVH |
| *Morina chinensis* | 101.9792 | 38.27083 | CVH |
| *Morina chinensis* | 102.3958 | 31.02083 | GBIF |
| *Morina chinensis* | 102.4792 | 30.89583 | CVH |
| *Morina chinensis* | 102.4792 | 35.1875 | CVH |
| *Morina chinensis* | 102.5625 | 35.22917 | CVH |
| *Morina chinensis* | 102.8125 | 31.02083 | CVH |
| *Morina chinensis* | 102.8542 | 31.02083 | CVH |
| *Morina chinensis* | 102.8542 | 34.39583 | CVH |
| *Morina chinensis* | 102.8958 | 30.9375 | CVH |
| *Morina chinensis* | 102.9792 | 33.60417 | CVH |
| *Morina chinensis* | 103.1458 | 37.3125 | GBIF |
| *Morina chinensis* | 103.1875 | 37.3125 | GBIF |
| *Morina chinensis* | 103.5625 | 32.64583 | CVH |
| *Morina chinensis* | 103.6042 | 32.8125 | GBIF |
| *Morina chinensis* | 103.6875 | 33.02083 | CVH |
| *Morina chinensis* | 103.9792 | 34.39583 | CVH |
| *Morina chinensis* | 99.6875 | 27.8125 | GBIF |
| *Morina chinensis* | 99.6875 | 34.60417 | GBIF |
| *Morina chinensis* | 99.72917 | 29.39583 | CVH |
| *Morina kokonorica* | 100.3958 | 34.39583 | GBIF |
| *Morina kokonorica* | 100.6042 | 31.39583 | GBIF |
| *Morina kokonorica* | 100.6875 | 31.39583 | GBIF |
| *Morina kokonorica* | 100.8125 | 32.8125 | GBIF |
| *Morina kokonorica* | 100.8125 | 36.72917 | GBIF |
| *Morina kokonorica* | 102.6875 | 30.85417 | GBIF |
| *Morina kokonorica* | 102.9375 | 31.0625 | CVH |
| *Morina kokonorica* | 103.1875 | 34.22917 | CVH |
| *Morina kokonorica* | 103.3125 | 32.97917 | GBIF |
| *Morina kokonorica* | 81.1875 | 30.3125 | GBIF |
| *Morina kokonorica* | 84.02083 | 29.77083 | GBIF |
| *Morina kokonorica* | 84.10417 | 32.3125 | GBIF |
| *Morina kokonorica* | 85.22917 | 29.35417 | GBIF |
| *Morina kokonorica* | 85.3125 | 28.89583 | GBIF |
| *Morina kokonorica* | 85.97917 | 28.64583 | CVH |
| *Morina kokonorica* | 87.10417 | 28.6875 | GBIF |
| *Morina kokonorica* | 87.1875 | 29.3125 | GIBF |
| *Morina kokonorica* | 87.8125 | 28.39583 | CVH |
| *Morina kokonorica* | 88.85417 | 33.1875 | CVH |
| *Morina kokonorica* | 89.14583 | 27.72917 | CVH |
| *Morina kokonorica* | 89.97917 | 31.39583 | CVH |
| *Morina kokonorica* | 90.6875 | 29.39583 | GBIF |
| *Morina kokonorica* | 91.0625 | 29.6875 | CVH |
| *Morina kokonorica* | 91.10417 | 29.6875 | GBIF |
| *Morina kokonorica* | 91.10417 | 30.47917 | GBIF |
| *Morina kokonorica* | 91.14583 | 29.6875 | CVH |
| *Morina kokonorica* | 91.1875 | 29.72917 | CVH |
| *Morina kokonorica* | 91.27083 | 29.39583 | CVH |
| *Morina kokonorica* | 91.27083 | 30.10417 | CVH |
| *Morina kokonorica* | 91.3125 | 29.89583 | GBIF |
| *Morina kokonorica* | 91.5625 | 32.0625 | CVH |
| *Morina kokonorica* | 91.64583 | 28.6875 | CVH |
| *Morina kokonorica* | 91.85417 | 27.9375 | CVH |
| *Morina kokonorica* | 91.89583 | 28.97917 | CVH |
| *Morina kokonorica* | 92.1875 | 29.6875 | GBIF |
| *Morina kokonorica* | 92.35417 | 31.47917 | CVH |
| *Morina kokonorica* | 92.60417 | 29.10417 | GBIF |
| *Morina kokonorica* | 92.97917 | 31.85417 | CVH |
| *Morina kokonorica* | 93.6875 | 31.47917 | GBIF |
| *Morina kokonorica* | 94.0625 | 31.9375 | CVH |
| *Morina kokonorica* | 94.14583 | 29.64583 | CVH |
| *Morina kokonorica* | 94.39583 | 32.97917 | GBIF |
| *Morina kokonorica* | 94.4375 | 29.6875 | CVH |
| *Morina kokonorica* | 94.64583 | 31.5625 | CVH |
| *Morina kokonorica* | 95.22917 | 31.5625 | CVH |
| *Morina kokonorica* | 95.60417 | 31.39583 | GBIF |
| *Morina kokonorica* | 95.77083 | 34.22917 | CVH |
| *Morina kokonorica* | 95.8125 | 29.89583 | GBIF |
| *Morina kokonorica* | 95.8125 | 30.72917 | CVH |
| *Morina kokonorica* | 95.8125 | 31.14583 | CVH |
| *Morina kokonorica* | 96.5625 | 31.89583 | CVH |
| *Morina kokonorica* | 96.60417 | 31.22917 | CVH |
| *Morina kokonorica* | 96.64583 | 31.22917 | CVH |
| *Morina kokonorica* | 96.6875 | 32.8125 | GBIF |
| *Morina kokonorica* | 96.77083 | 29.52083 | CVH |
| *Morina kokonorica* | 96.77083 | 33.0625 | CVH |
| *Morina kokonorica* | 96.89583 | 30.10417 | GBIF |
| *Morina kokonorica* | 96.89583 | 33.0625 | CVH |
| *Morina kokonorica* | 96.97917 | 32.10417 | GBIF |
| *Morina kokonorica* | 97.02083 | 32.0625 | CVH |
| *Morina kokonorica* | 97.27083 | 32.10417 | GBIF |
| *Morina kokonorica* | 97.3125 | 32.10417 | GBIF |
| *Morina kokonorica* | 97.3125 | 33.39583 | CVH |
| *Morina kokonorica* | 97.5625 | 32.02083 | CVH |
| *Morina kokonorica* | 97.60417 | 31.35417 | CVH |
| *Morina kokonorica* | 97.8125 | 29.6875 | GBIF |
| *Morina kokonorica* | 98.1875 | 31.47917 | GBIF |
| *Morina kokonorica* | 99.8125 | 28.89583 | GBIF |
| *Morina kokonorica* | 99.89583 | 36.97917 | CVH |
| *Morina kokonorica* | 99.97917 | 31.60417 | GBIF |
